# Iliotibial Band Behavior Assessed Through Tensor Fasciae Latae Electromyographic Activity with Different Foot Orthoses in Recreational Runners According to Foot Type: A Cross-Sectional Study

**DOI:** 10.3390/jfmk10030237

**Published:** 2025-06-23

**Authors:** Ruben Sanchez-Gomez, Álvaro Gómez Carrión, Ismael Ortuño Soriano, Paola Sanz Wozniak, Ignacio Zaragoza García, Fatma Ben Waer, Cristina Iona Alexe, Dan Iulian Alexe

**Affiliations:** 1 Nursing Department, Faculty of Nursing, Physiotherapy and Podiatry, Universidad Complutense de Madrid, 28040 Madrid, Spain; alvgom25@ucm.es (Á.G.C.); iortunos@ucm.es (I.O.S.); paolsanz@ucm.es (P.S.W.); izaragoz@ucm.es (I.Z.G.); 2 Instituto de Investigación Sanitaria Hospital Clínico San Carlos (IdISSC), 28040 Madrid, Spain; 3 Clínica Pododinámica, 28005 Madrid, Spain; 4 Grupo Invecuid, Instituto de Investigación Sanitaria imas12, 28041 Madrid, Spain; 5 Research Laboratory Education, Motricité, Sport et Santé, LR19JS01, High Institute of Sport and Physical Education of Sfax, University of Sfax, Sfax 3029, Tunisia; fatmaelwaer123@gmail.com; 6 Department of Physical Education and Sports Performance, “Vasile Alecsandri” University of Bacău, 600115 Bacău, Romania; alexe.cristina@ub.ro; 7 Department of Physical and Occupational Therapy, Faculty of Movement, Sports and Health, Sciences, “Vasile Alecsandri” University of Bacău, 600115 Bacău, Romania; alexedaniulian@ub.ro

**Keywords:** iliotibial band syndrome, foot orthoses, recreational runners, foot type, therapeutic alternative

## Abstract

**Background:** Iliotibial band syndrome (ITBS) through the tensor fascia latae (TFL) is a well-known pathology among runners whose etiology is not completely clear, nor is the effectiveness of plantar insoles for different types of feet known well enough for them to be considered a possible approach for this issue. **Objective:** to understand how foot type and foot orthotics may influence the electromyographic (EMG) activity of the TFL. **Methods:** A total of 41 healthy recreational runners (mean age 32.66 ± 3.51) were recruited for the present cross-sectional study, categorizing them as neutral (NEUg = 15), supinators (SUPg = 15), and pronators (PROg = 11) according to the foot postural index, over a period of 11 months. The EMG of the TFL was measured using a surface electromyograph device while they ran on a treadmill at a constant speed of 9 km/h for 3 min, randomly using supinating (SUP), pronating (PRO), or heel lift (TAL) insoles of 5 mm each one, compared to the baseline condition (SIN). The intraclass correlation coefficient (ICC) was performed to check the reproducibility of the tests, pairwise comparisons with Bonferroni adjustment were made, and to test the differences between measurements, the Friedman test was performed. **Results**: The Shapiro–Wilk test indicated a normal distribution of the sample (*p* > 0.05). Almost all obtained results showed a “perfect reproducibility” close to one; a significant statistical increase was observed in the mean EMG values from NEUg (87.58 ± 4.81 mV) to SUPg (97.17 ± 4.3 mV) (*p* < 0.05) during SIN+ basal condition. Additionally, there was a statistical reduction from SIN (87.58 ± 4.81 mV) vs. PRO (74.69 ± 3.77 mV) (*p* < 0.001) in NEUg and from SIN (97.17 ± 4.3 mV) vs. PRO (90.96 ± 4 mV) (*p* < 0.001) in SUPg. **Conclusions**: The SUPg exhibited increased activation of TFL fibers compared to the NEUg, likely due to the biomechanical demands associated with a supinated foot type. In contrast, the use of PRO appeared to promote relaxation of the TFL fibers by inducing internal rotation of the lower limb. Based on these preliminary results from a cross-sectional study in a healthy population, it is recommended to assess foot type when addressing ITBS and to consider the use of PRO as a complementary therapeutic strategy alongside conventional treatments.

## 1. Introduction

Iliotibial band syndrome (ITBS) is a common overuse pathology among recreational runners, characterized by swelling and acute pain in the lateral region of the knee. Its symptoms typically worsen during knee transitions in flexion–extension movements and are intimately related to the initial phase of running activity, commonly occurring during single-leg stance [1,2]. It has an estimated prevalence of 5% to 12%, making it one of the most common injuries among this type of athlete after Achilles tendinopathy [2].

Anatomically, the iliotibial band (ITB), also known as Maissiat’s band, is the distal prolongation of the tensor fasciae latae (TFL) muscle, which originates from the anterior area of the iliac crest and receives one of the insertions of the gluteus maximus (Gmax) [3,4]. It runs superficial to the vastus lateralis and inserts on Gerdy’s tubercle (GTb) of the tibia, partially to the supracondylar ridge of the lateral femur, and on the lateral area of the patella, serving as the most important attachment points [5]. Due to its location, the ITB collaborates with the gluteus group in hip abduction movements. Depending on the flexion grades of the hip and the levels of fiber activation of the Gmax and TFL, either internal or external rotation of the lower limb can occur. It remains a relative mystery which of them has the main predominance [5]. On the other hand, the ITB has been identified as a stabilizer of the pelvis and knee during single-leg circumstances, such as the swing phase of walking and running gait [6].

The etiology of ITBS is multifactorial and somewhat confused in the current literature because the TFL and Gmax have a critical influence on its pathophysiology. Additionally, it is a double-jointed structure, crossing both the hip and knee, which further complicates understanding [5]; the latest theories, supported by sonographic [7] and cadaveric studies (4) that used magnetic resonance imaging, discard the classical theories of backward displacement and friction [8,9,10] and argued that when the knee is flexed past 30°, the ITB compresses medially over the lateral femoral epicondyle. This repetitive situation could generate inflammation and an over-compression stress syndrome between the interposed layer of richly innervated and vascularized fat pad and the anchored ITB fibrous strands on the lateral femur [4,11]. Several studies have found a relationship between greater adductor moments of the hip, associated with weakness in the hip abductors, specifically the GMax or TFL, and the presence of ITBS in runners [2,8,12,13].

However, it remains unclear whether foot supination or pronation could alter the muscular activity of TFL during running, which is the principal assistant of the ITB [14,15], since it is known how the foot position affects lower limb alignment. In that sense, it has been shown that internal rotation of the tibia is always combined with internal rotation, valgus, and adduction of the knee, linked to foot pronation movements [16], which indicates the dynamic relationship between these structures, whereas external rotation has been associated with varus and abduction [17], but there is no consensus in the literature regarding which mechanism is most likely to cause the ITB injury: some papers advocate that ITBS is caused by internal rotation and/or adduction mechanisms [18,19], whereas others argue that it is external rotation and/or abduction movements that trigger it [17,20]. In addition, it has even been suggested that lower limb discrepancy is another etiology of ITBS [21], although there are currently no references regarding the orthopedic approach to improve this pathology. Any hypersupinatory, hyperpronatory, or limb-length discrepancy-related imbalances, induced artificially by a foot orthosis or by the patient’s own anatomical foot deviation, can generate a response throughout the lower limb kinetic chain [22,23], thereby affecting the ITB. Therefore, an orthotic insole that compensates for these alterations could thus be hypothesized to help balance and improve ITB function.

Since it is known that any muscle overuse would produce increased muscle activity in the values of a given surface electromyography EMG [24], it would be possible to determine which lower limb movements through the foot increase ITB activity and which decrease it. However, given that due to its tissue characteristics, the ITB lacks myocytes with electrical activity to be measured, its activity could be inferred through the measurement of the TFL muscle activity by EMG. In that sense, EMG has been reported as a valid tool to assess the muscular activity of hip muscles such as the Gmax and TFL in different conditions [25].

The lack of consensus in the current literature regarding the precise role of the ITB in knee stability and its implications in the development of ITBS in runners [5,6] together with the absence of evidence demonstrating the influence of foot type, alignment, and lower limb discrepancies on the ITB, has motivated the design of the present study. In addition, most available treatments do not target lower limb biomechanics as a central therapeutic goal to address the structural origins of the syndrome [18]; instead, most interventions primarily focus on strengthening the hip musculature, including the gluteal group, abdominals, and the thigh muscles [26] and standard electrotherapeutic interventions aimed at pain relief, including therapeutic ultrasound and shock-wave therapy [27].

Therefore, the present study aims to investigate ITB behaviour through the EMG activity of the TFL while wearing different orthoses, including pronation (PRO), supination (SUP), and heel lift (TAL) compared to basal condition with standard sports shoes (SIN), during running activity in neutral (NEUg), supinated (SUPg), and pronated (PROg) participants. Since the internal rotation of the lower limb involves the shortening of the origin-insertion unit of the TFL muscle fibers, and this internal rotation will be promoted by foot pronation with the use of PRO, it is hypothesized that internal rotation induced by PRO could decrease the EMG signal of the TFL in all groups, thereby reducing tension on the ITB and providing a new approach to ITBS management.

## 2. Materials and Methods

The present cross-sectional observational analytic study was approved by a specific Ethics Committee (C.I. 22/740-E) on 22 December 2022. All ethical and human concerns were respected according to the “Declaration of Helsinki 1964”. All participants were informed about the research, and they signed a consent agreement.

### 2.1. Sample Size and Study Design

The convenience and the total sample size were calculated by the specific statistical unit. The aim was to compare the differences in TFL’s EMG activity among participants with NEUg, SUPg, and PROg foot positions, and to study the changes suffered while wearing SUP, PRO, and TAL orthoses versus SIN. Previous studies [28] of the Gmax EMG activity were recorded, obtaining 97.61 ± 50.43 vs. 42.99 ± 25.89 millivolts (mV) (mean ± SD) (*p* < 0.001) for concentric and eccentric activity, respectively. Considering a statistical power of 80%, a 95% confidence interval (CI), β = 20%, and α = 0.05, it was estimated that 12 subjects per group were needed to conduct the present study to detect a difference in the mean EMG of 0.75 mV using ANOVA for independent samples. Considering potential loss to follow-up, we established a final sample size of at least 12 subjects per group. The Strengthening the Reporting of Observational Studies in Epidemiology (STROBE) guidelines were followed [29], and non-probability purposive sampling techniques were followed throughout this cross-sectional analytic observational study, which was conducted between February 2023 and January 2024. This was a single-blind study in which the subjects were unaware of the variable being applied at each moment. The variables were assigned through a simple randomization process using the OxMaR^®^ software v2019.

### 2.2. Participants

The following criteria were used to select the sample, as conducted by the RSG researcher: General inclusion criteria: (1) healthy women and men between 18 and 35 years old, (2) recreational rearfoot strike pattern runners with 3 training days per week and with at least 1 year of experience, and (3) to have experience on run over a treadmill. General exclusion criteria: (1) to have any pain and/or any lower limb or foot injury at the time of the test or 1 year ago, (2) to have any restriction pathology on the lower limb far from valid values [30,31], and (3) to be under any medication effects at the time of the test. Specific inclusion criteria [32] for (1) NEUg: foot posture index (FPI) with scores between 0 and 5; (2) for PROg: FPI with scores between +6 and +9; and (3) for SUPg: FPI with scores between −1 and −4. Specific exclusion criteria: to have more than FPI indicated values for each differentiated group. Body mass index (BMI) = weight (kg)/height (m^2^) was calculated, and foot sizes between 38 and 42 were required, avoiding hypothetical bias on data recollection, or interpreting the results. The high variability in BMI, as well as the different running techniques linked to experience or other physical characteristics, could interfere with the reproducibility of the results, as muscle strength could be affected by these factors.

### 2.3. Instruments and Assessments

A NeuroTrac Simplex Plus^®^ (Verity Medical Ltd., Braishfield, UK) electromyographic surface EMG device, validated in previous studies [33,34], was used to assess the muscle contraction activity of the TFL fibers during running trials. The device had a recording range of 0.2 mV to 2000 mV with an accuracy of 4% of the reading from mV ± 0.3 mV up to 200 Hz. It featured a bandpass filter from 18 Hz ± 4 Hz to 370 Hz ± 10 Hz for readings below 235 mV, and a sensitivity of 0.1 mV root mean square (RMS). Its wireless connection range was 10 m via Bluetooth. To detect the signal of the TFL electrical action, self-adhesive circular surface electrodes measuring 30 mm in diameter and made of high-quality hydrogel and conductive carbon film were utilized. The signal was captured by the receiver module and automatically filtered by the NeuroTrac^®^ software v 5.0.121. Subsequently, it was transmitted via a secure unidirectional radioelectric connection to the computer, where the software digitally transformed it to generate activity pattern data for each electrode.

### 2.4. Materials

**Figure 1:**
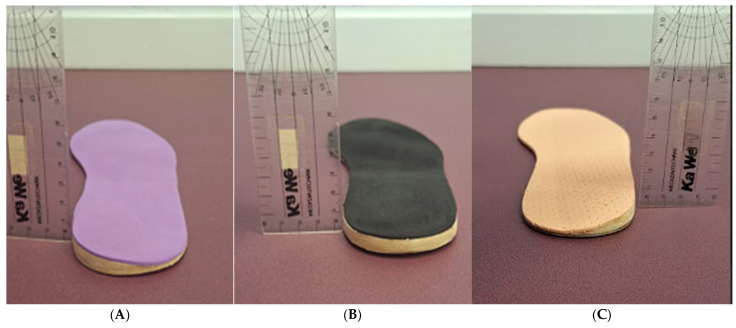
Orthoses used in the present study, with the scale set at 5 mm thickness for each one: (**A**) detail of Supination orthotic = SUP.; (**B**) detail of Heel lift orthotic = TAL; (**C**) detail of Pronation orthotic = PRO.

A flat sheet of ethylene-vinyl acetate (EVA) was used to create standard orthoses for the participants. It had a semi-rigid density and was 1 mm thick, without any orthotic element that could interfere with the normal biomechanical behaviour of the foot. The SUP, PRO, or TAL elements were then added. SUP featured a hard, rigid posting wedge of 5 mm EVA thickness placed on the medial rearfoot of the orthotic (Figure 1A), while PRO had the same posting but placed on the lateral side (Figure 1C). TAL featured a flat neutral brick of 5 mm EVA thickness [35] placed on the rearfoot surface (Figure 1B). A thin layer of 1 mm soft EVA covered all the orthoses. To avoid hypothetical biases and imbalances, the same flat insole with identical characteristics was placed on the contralateral foot, except for TAL, which was placed on the contralateral side where we aimed to assess the EMG activity, creating an “artificial” lower limb discrepancy. The laboratory that produced the orthoses was an external company in the Orthoses Industry (Termofeet^®^ SL, Madrid, Spain) and remained blinded throughout all stages of the study. The neutral sports shoes used were ‘New Feel PW^®^ 10 M medium grey’ (ref. number: 20182022).

### 2.5. Procedure

The specialist in sports podiatry and researcher (RSG) took measurements of the participants. To determine the optimal location of the sensors for assessing the EMG of the TFL, the researcher instructed participants to lie on their dominant lateral side on the clinical bed, placing the non-dominant leg above as it was the weaker leg. Then, participants were asked to move their thighs toward the ceiling, resisting the pressure applied by the researcher at the knee joint, to make the muscular belly of the TFL visible and mark it. Once this was completed, two sensors were placed according to the protocol described by the European Project “Surface ElectroMyoGraphy for the Non-Invasive Assessment of Muscles” (SENIAM: http://www.seniam.org (accessed on 8 January 2023). with a longitudinal orientation aligned with the muscle belly fibers of the TFL, and placed 3 cm apart from each other. The neutral electrode was positioned outside the muscle belly of the evaluated muscle, on the vastus lateralis muscle, following the manufacturer’s instructions. Subsequently, participants performed a maximal abduction of the hip with knee flexion against the resistance provided by the researcher for 5 s to establish the maximal voluntary contraction (MVC), calibrate the device, and normalize the signal amplitudes of each EMG data.

#### Running Test

An automatic motorized treadmill (Domyos^®^ T520) was used for the running test. Each participant ran for 3 min at 5.7 km/h on the treadmill before the test, using the different orthoses and SIN, as an acclimatization period to the device, aimed at reducing potential biases associated with the use of new materials. Subsequently, all running tests were conducted at a speed of 9 km/h [36] for 3 min for all participants to standardize the intensity and minimize the influence of varying speeds on muscle activation that could interfere with the EMG signal [37]. A simple randomized sequence was created using OxMaR^®^ software to perform the test for the four described conditions (SUP, PRO, SIN, TAL) on the same day for each participant. All participants ran under the four different conditions. The mean EMG activity of the TFL for each condition was recorded for 30 s, three times, resulting in a total of 12 tests per participant. There was a 5 min rest period as a wash-out period between each trial to allow for adequate recovery [38]. All tests were conducted with the same orthotic placed on the contralateral foot, except for the TAL condition, where it was placed on the dominant foot without any compensation on the contralateral side. This approach aimed to disrupt the stability of the weaker contralateral TFL leg.

### 2.6. Statistical Analysis

The normality of the sample was calculated using the Shapiro–Wilk test, considering normal distribution if *p* > 0.05. To test the differences between sociodemographic characteristics, an ANOVA test was performed. To determine the real magnitude of the relationship or difference between means, Cohen’s d was calculated. To evaluate differences between conditions and participants, pairwise comparisons with Bonferroni adjustment were conducted. To assess differences between measurements, the Friedman test was performed. Statistical analyses were performed using IBM^®^ SPSS^®^ Statistics version 29 (2022), with a significance level set at α = 0.05.

Anthropometric and demographic characteristics are managed as means and standard deviations (±SD). The within-day trial-to-trial intraclass correlation coefficient (ICC) and standard error of measurement (SEM) were calculated [39] for all 3 groups of subjects under 4 different conditions to check the reliability of the research; the SEM was calculated to find the minimum detectable change (MDC) for all measurements. The obtained results of the ICC were categorized according to Landis and Koch [4] classification, where the ICCs would be slight if it shown <0.20; fair if 0.20–0.40; moderate if 0.41–0.60; substantial if 0.61–0.80; and almost perfect agreement if 0.81–1.00, considering ICCs of ≥0.81 as the better values to achieve the optimal scientific validity of the results.

## 3. Results

**Figure 2:**
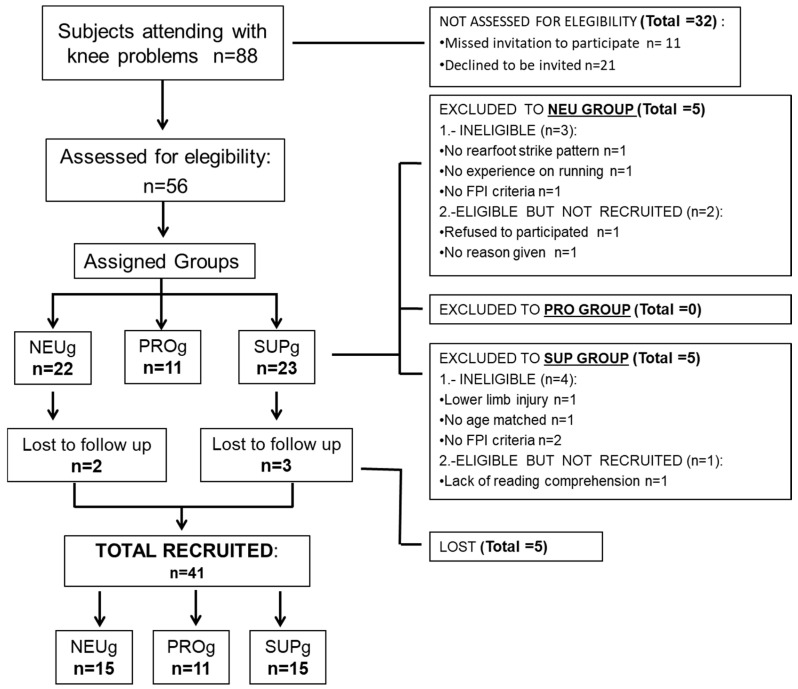
Participant flow chart. Abbreviations: NEUg = neutral group; SUPg = supinated group; PROg = pronated group.

A total of 56 healthy and recreational runner participants (36 female and 20 male) from a podiatry and sports orthopedics clinic were recruited to participate in the study following non-probability purposive sampling; 22 were categorized as NEUg, 11 as PROg, and 23 as SUPg. Ten participants did not meet the inclusion criteria, and five were lost to follow-up. Finally, 41 participants (15 NEUg, 15 SUPg, and 11 PROg) were enrolled in the study (Figure 2). The anthropometric characteristics of the subjects are presented in Table 1, including differences between groups. Table 2 shows the effect sizes calculated using Cohen’s d. No missing data were observed in any condition after the sample was selected and filtered.

The ANOVA analysis indicated that there were significant or highly significant differences in the variables analyzed among the groups studied.

Overall, the effect sizes of Cohen’s d suggested that there were significant differences in variables such as age, weight, and foot length, while BMI remained relatively constant across the groups.

The Shapiro–Wilk test indicated a normal distribution of the sample (*p* > 0.05). The ICC and SEM were conducted to evaluate the reliability of the EMG muscle data recorded during the test, as shown in Table 3. The majority of the recorded data achieved a value of one or approached one, indicating ‘perfect reliability’ according to the reference [39]; Table 4 shows the effect sizes calculated using Cohen’s d.

In all interventions (SIN, SUP, PRO, and TAL), ANOVA analysis showed a strong statistically significant differences were found among the three groups. At least one group has a mean that is significantly different from the others.

The results of Cohen’s d showed very large effect sizes in several comparisons, reflecting substantial differences in muscle activity (mV) between groups during various interventions; these marked differences—especially effect sizes above d = 2—indicated that the neuromuscular response to the interventions varied considerably among the groups. The SUPg group consistently appeared to exhibit greater muscle activation, in contrast to PROg and NEUg.

Table 5 shows the comparisons and *p*-values of the baseline EMG activity of the TFL in each group without any type of intervention.

**Figure 3:**
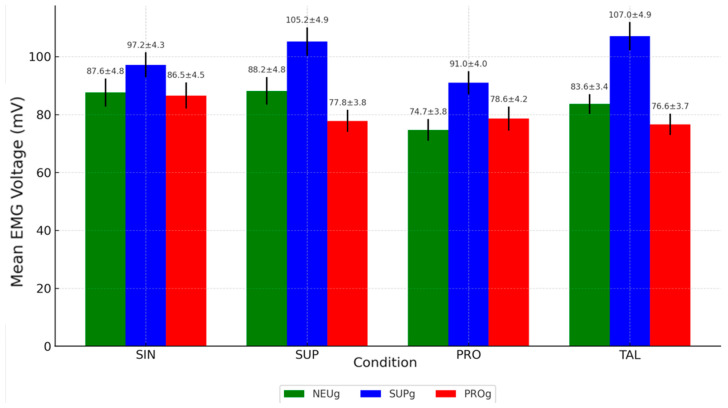
Mean EMG activity of TFL during running activity by condition and group.

The comparative EMG data of the TFL running for each group under four different conditions are presented in Table 6, along with their respective *p*-values. Figure 3 graphically represents the behavior of these values under the different interventions across the various groups.

Figure 3 showed that individuals with a tendency toward supination and without any intervention (SIN) had higher mean EMG values in their TFL activity (97.17 ± 4.3 mV) compared to neutral (87.58 ± 4.81 mV; *p* < 0.05) and pronated (86.55 ± 4.5 mV; *p* < 0.05) participants.

### 3.1. Results for Neutral Group Foot Type

The NEUg showed a significantly lower EMG activity in the PRO condition (74.69 ± 3.77 mV) compared to SUP (88.16 ± 4.76 mV), with a *p*-value of <0.001, indicating a notable reduction in muscle activation during running using PRO. No other statistically significant differences were found among the conditions in this group.

### 3.2. Results for Supinated Group Foot Type

The SUPg exhibited multiple statistically significant differences:

- EMG activity was significantly higher in the SUP (105.22 ± 4.9 mV) and TAL (107.02 ± 4.88 mV) conditions compared to the SIN condition (97.17 ± 4.3 mV), both with *p*-values < 0.05.
- Both SUP and TAL conditions also showed significantly higher values than PRO (90.96 ± 4 mV), with *p*-values < 0.05 and <0.001, respectively.

These findings indicate that SUP and TAL interventions led to increased TFL activation in this group, whereas PRO significantly decreased the EMG values.

### 3.3. Results for Pronated Group Foot Type

In the PROg, although the values obtained for each intervention were lower than those in the SIN, there were no statistically significant differences in EMG activity between any of the four conditions.

This is the bar chart showing the mean EMG voltage values for each condition (SIN, SUP, PRO, TAL) across the three groups (NEUg, SUPg, PROg):

Moderate EMG activation levels were observed in NEUg (red bars), ranging from 74.69 ± 3.77 mV in the PRO condition to 88.16 ± 4.76 mV in the SUP condition. The EMG activity in the PRO condition was significantly lower than in SUP (*p* < 0.001). This reduction may indicate a decreased role of the TFL under pronated conditions in individuals with neutral foot posture.

SUPg (blue bars) consistently showed the highest TFL activation levels, particularly under the SUP condition (105.22 ± 4.90 mV) and the TAL condition (107.02 ± 4.88 mV). These values were markedly higher than those in the SIN condition (97.17 ± 4.30 mV) and PRO condition (90.96 ± 4.00 mV), both with statistically significant differences.

PROg (green bars) showed the lowest and most stable EMG values, with means ranging from 76.62 ± 3.70 mV (TAL) to 86.55 ± 4.50 mV (SIN). The lack of statistically significant differences between conditions (all *p* = 1) suggests that in pronated individuals, TFL activity remains relatively unchanged regardless of the running condition.

## 4. Discussion

The goal of the present study was to investigate how the muscle activity of the ITB could be influenced by foot type (NEUg, SUPg, and PROg) when using three different types of plantar orthotics (SUP, PRO, TAL). This aimed to elucidate the potential causes of ITBS associated with lower limb biomechanics during running. Additionally, the etiology of ITBS remains unclear, leading to numerous diagnostics and treatments that have lacked strong empirical support for years [2,9,19]. By knowing and thereby controlling the effect of foot position and its biomechanical behavior on ITB activity, it may be possible to improve the recovery time of patients suffering from this classic injury.

If considering the structural anatomy of TFL in connection with ITB and its force’s vector of insertion onto GTb [14,40], the internal rotation of the lower limb may induce relaxation of the ITB due to the shortened origin-insertion distance of the TFL fibers with the external rotation having the opposite effect [3,19], which is associated with the swelling of its distal fibers on GTb as recent literature suggests, attributed to compression of the innervated fat pad against the lateral epicondyle [2,11]. In line with these assumptions, our results demonstrated that SUPg exhibited significantly higher EMG activity of TFL than NEUg or PROg; this suggests that increasing supinated movements at the foot, knee, or hip/femur complex may lead to greater compression of the fat pad at the GTb of ITB insertion as the space between the taut tendon and femoral condyle becomes narrower. This concept finds support in our study, wherein significant and substantial decreases in TFL EMG activity were observed in NEUg and SUPg when using PRO orthoses since these orthoses likely reduced muscle activity by bringing the origin and insertion points closer together—thus shortening the origin-insertion unit of the ITB, thanks to promoting internal rotation of the lower limb. Although PROg also exhibited a decrease, the reduction was not statistically significant, possibly due to the structural chronic internal rotation present in those participants, which may have obscured the effect of the orthoses. These promising results obtained in a healthy population may have potential future applicability in pathological populations presenting with tibial varus or marked external hip rotation.

On the other hand, it is recognized that foot type can influence knee pathologies such as knee osteoarthritis, particularly in individuals with either pronated or supinated feet [41,42]; for instance, based on previous and extensive literature [43,44,45], an overpronated foot and/or flat foot, may lead to increase internal rotation kinematics of the lower limb; consistent with our findings, we observed that individuals with basal PROg exhibited significantly lower EMG activity of the TFL activity than basal NEUg and SUPg; however our findings contrast with a recent Oxford Model study that did not find any major differences in the tibia motion segment in individuals with symptomatic flexible flat foot [46]. It is worth noting that while our study was conducted in a healthy population, the results are inconsistent with those obtained in individuals with symptomatic flat foot in the Oxford Model study. Similarly, our results contradict those of another study that found no changes in kinematics between symptomatic and asymptomatic ITB runners [47]. These discrepancies or null findings may be attributed to several factors, including the lack of standardized protocols for assessment, differences between functional and quantitative techniques, variability in the study samples, effects of different surfaces, foot biomechanics not considered, or other muscular compensations.

Nowadays, there is extensive debate regarding whether ITBS is a cause or consequence of lower limb injuries, leading to unclear treatment strategies. Some authors [37,48,49,50] suggested that the progressive reduction in peak hip adduction angle during prolonged running activities may be linked to strategies aimed at reducing pain, whereas others have demonstrated increased tension in the TFL in individuals with ITBS [37], but overall, the present paper shows that considering internal rotation of the lower limb as synonym of hip adduction, reached by the foreign effect of PRO, TFL exhibited a decrease in EMG activity in SUPg and PROg; moreover, TFL exhibited the opposite and expected behaviour when SUP orthotics were worn by SUPg, accompanied by an statistical increase in EMG data. This increase was likely due to external rotation, which tightens its fibers and lengthens the origin-to-insertion distance, in line with previous authors who had hypothesized similar results related to neuromuscular effects [51,52]. However, the obtained results of the present study cannot be directly compared with previous research, as to date, no studies have examined the behavior of hip musculature in relation to foot type or the use of custom-made foot orthoses.

Despite numerous therapeutic strategies proposed to improve ITBS [53,54,55,56], there is limited literature considering orthopedic insoles as an option to complement conventional treatments [2] by targeting muscular fiber orientation, as has been explored in the context of patellofemoral pathologies [56]. In that sense, we have demonstrated that using PRO resulted in a significant decrease in TFL’s EMG activity in NEUg and SUPg, and without taking into account that the results would likely be even more promising if custom-made insoles were used for each patient, incorporating these elements that enhance TFL activity [57,58]. To our knowledge, there are no previous references specifically exploring these benefits on knee musculature; only a few studies [49,52] have linked internal rotation with hip adduction, suggesting that a reduction in hip adduction may be a significant indicator of ITBS and that hip muscle strengthening programs should be developed to compensate for the functional impairment.

Finally, some authors have hypothesized that lower limb discrepancies could contribute to various lower limb injuries [21,59] such as lumbar pain when the dysmetria is greater than 5 mm [21] or knee problems [56,60]. The pelvic tilt generated by lower limb discrepancies modifies the tension of hip muscles [2] including TFL. Although our participants did not have any lower limb length discrepancies, when we “artificially” altered their pelvic balance with TAL in the contralateral leg, our results demonstrated that TFL activity worsened (increasing EMG activity) in SUPg when compared to SIN, similar to obtained results by Benito et al. [35]; therefore, this finding highlights the direct relationship between these variables and emphasized the control role of the hip muscles as we known at the time [3,28,53,55].

### Limitations and Future Lines of Investigation

All mean EMG results should be interpreted with caution due to the high sensitivity of the EMG device used, variations in the maximal voluntary isometric contraction test used to calibrate the signal may exist between individuals, and the possible fatigue effect on the participants. Additionally, this was a single-blind study in which the participants were unaware of the type of insole they were using, while the examiners were aware of the insole types assigned to each participant.

The authors are not aware of any preventive effects that the use of insoles may have in avoiding the development of ITBS. The limitation of our study to a healthy population restricted our ability to investigate potential relationships between ITBS, lower limb discrepancies, and TFL activity in symptomatic individuals. The present research was specifically designed to evaluate the preliminary effects of insoles on the TFL in a healthy population, without the interference of pain or other potential biomechanical compensations in the leg or hip. As the findings were obtained from a cross-sectional design, they can only serve to generate hypotheses. Therefore, future randomized controlled trials and longitudinal studies involving injured or symptomatic populations are recommended to elucidate these issues. Further research in such populations is also needed to assess the potential of insoles to reduce pain and improve functional outcomes.

The EMG values represent the entire running gait cycle; therefore, future studies should aim to define TFL activity during the different phases of this cycle. In addition, the results were obtained at a specific treadmill speed and recorded over a short period of time. Future studies should investigate whether both or other variables, such as type of surface or environment, could influence the strength, fatigue, and energy consumption capabilities of muscles, hypothetically in a directly proportional manner.

## 5. Conclusions

This study provides new insights into the functional relationship between foot posture, lower limb biomechanics, and TFL activity during running, which may contribute to a better understanding of the etiopathogenesis of ITBS. The preliminary results of this observational, analytical study in a healthy population demonstrate that TFL muscle activity varies according to foot type and orthotic intervention, supporting the hypothesis that foot-induced biomechanical alterations indirectly influence ITB activity. Specifically, the use of PRO promoted an internal rotation of the lower limb, leading to a significant reduction in TFL EMG activity—especially in SUPg and NEUg foot types—suggesting a potential therapeutic effect by relieving ITB tension. In contrast, the addition of TAL increased TFL activation, possibly due to an induced artificial imbalance, thus reinforcing the role of the hip musculature in stabilizing the lower limb. These findings highlight the relevance of assessing foot posture and biomechanics in the management of ITBS and support the consideration of PRO insoles as a corrective strategy for runners with neutral or supinated foot types. Although preliminary and based on healthy individuals, these results open the possibility for future longitudinal and/or cross-sectional studies on an injured population to explore the role of foot orthoses as part of a comprehensive, biomechanically informed, and complementary treatment protocol for ITBS.

## Figures and Tables

**Table 1:** Subject demographics description.

	Total Population N = 41	Neutral Group (NEUg)	Supinated Group (SUPg)	Pronated Group (PROg)	
		n = 15	n = 15	n = 11	
Variable	Mean ± SD	Mean ± SD	Mean ± SD	Mean ± SD	F *p*-Value
	(95% CI)	(95% CI)	(95% CI)	(95% CI)	
	%	%	%	%	
age (years)	32.66 ± 3.51	33 ± 3	36 ± 7	29 ± 4	7.62 *p* < 0.001 **
	(31.76–33.56)	(31.73–34.27)	(33.03–38.97)	(27.02–30.98)	
height (cm)	170 ± 1.43	168.87 ± 4	170 ± 2	171.72 ± 3	5.95 *p* < 0.001 **
	(169.64–170.36)	(167.18–170.56)	(169.16–170.84)	(170.2–173.2)	
weight (kg)	67 ± 3.17	61.4 ± 6	65.3 ± 2	67 ± 7	3.66 *p* < 0.05 *
	(66.19–67.81)	(58.86–63.94)	(64.46–66.14)	(63.53–70.47)	
foot size (Es)	41.2 ± 0.9	41.2 ± 2.2	42.1 ± 1.8	40.3 ± 1.2	4.56 *p* < 0.001 **
	(40.97–41.43)	(40.27–42.13)	(41.34–42.86)	(39.71–40.89)	
BMI (kg/m^2^)	20.24 ± 0.19	20.3 ± 1.76	20.02 ± 2.04	20.4 ± 0.4	4.2 *p* < 0.05 *
	(20.2–20.28)	(19.56–21.04)	(19.16–20.88)	(20.21–20.59)	

Abbreviations: SD = standard deviation; CI = confidence interval; FPI = foot posture index; BMI = body mass index; Es = number according to European mode size; cm = centimeters; kg = kilograms; m^2^ = square meters. F = ANOVA: analysis of variance. *p*-value = level of significance; *p* < 0.05 * (with a 95% confidence interval) was considered statistically significant; *p* < 0.001 ** (with a 95% confidence interval) was considered strongly statistically significant.

**Table 2:** Subject Demographics Cohen’s d effect size.

Variable	Group 1	Group 2	Cohen’s d
Age	NEUg	SUPg	−0.557
Age	NEUg	PROg	1.159
Age	SUPg	PROg	1.179
Height	NEUg	SUPg	−0.357
Height	NEUg	PROg	−0.788
Height	SUPg	PROg	−0.697
Weight	NEUg	SUPg	−0.872
Weight	NEUg	PROg	−0.87
Weight	SUPg	PROg	−0.356
Foot Size	NEUg	SUPg	−0.448
Foot Size	NEUg	PROg	0.486
Foot Size	SUPg	PROg	1.141
BMI	NEUg	SUPg	0.147
BMI	NEUg	PROg	−0.073
BMI	SUPg	PROg	−0.241

Abbreviations: NEUg = neutral group; SUPg = supinated group; PROg = pronated group; BMI: body mass index.

**Table 3:** Reliability ICC and SEM values of variables of three groups in SIN, SUP, PRO, and TAL interventions.

	Neutral Group (NEUg)			Supinated Group (SUPg)			Pronated Group (PROg)			
	n = 15			n = 15			n = 11			
Intervention	Mean (mV) ± SD (95% CI)	ICC 95% IC	SEM	Mean (mV) ± SD (95% CI)	ICC 95% IC	SEM	Mean (mV) ± SD (95% CI)	ICC 95% IC	SEM	F *p*-Value
		(Li-Ls)			(Li-Ls)			(Li-Ls)		
SIN	87.58 ± 4.81 (85.16–90)	1	0.574	97.17 ± 4.3 (95.35–98.99)	0.999	1.02	86.55 ± 4.5 (84.32–88.78)	0.988	5.12	22.07 *p* < 0.001 **
		(1–1)			(0.999–1)			(0.966–0.996)		
SUP	88.16 ± 4.76 (86.14–90.18)	1	0.536	105.22 ± 4.9 (103.14–107.3)	1	0.882	77.81 ± 3,8 (75.93–79.69)	0.998	1.74	154.01 *p* < 0.001 **
		(1–1)			(0.999–1)			(0.994–0.999)		
PRO	74.69 ± 3.77 (73.09–76.29)	0.999	1.028	90.96 ± 4 (89.27–92.65)	1	0.63	78.58 ± 4.2 (76.5–80.66)	0.998	2	65.29 *p* < 0.001 **
		(0.998–1)			(0.999–1)			(0.994–0.999)		
TAL	83.64 ± 3.4 (76.61–80.49)	1	0.684	107.02 ± 4.88 (107.2–111.16)	0.999	1.116	76.62 ± 3.7 (74.79–78.45)	1	0.332	203.86 *p* < 0.001 **
		(0.999–1)			(0.999–1)			(1–1)		

Abbreviations: SD = standard deviation; CI = confidence interval; Li = lower limit; Ls = upper limit; SEM = standard error of measurement; ICC = intraclass correlation coefficient; mV = millivolts; SIN = basal condition sport shoes; SUP = supinated orthoses; PRO= pronated orthoses; TAL = heel lift orthoses; F = ANOVA: analysis of variance. *p*-value = level of significance; *p* < 0.001 ** (with a 95% confidence interval) was considered strongly statistically significant.

**Table 4:** Cohen’s d effect size.

Intervention	Group 1	Group 2	Cohen’s d
SIN	NEUg	SUPg	−2.102
SIN	NEUg	PROg	0.22
SIN	SUPg	PROg	2.422
SUP	NEUg	SUPg	−3.532
SUP	NEUg	PROg	2.36
SUP	SUPg	PROg	6.126
PRO	NEUg	SUPg	−4.186
PRO	NEUg	PROg	−0.984
PRO	SUPg	PROg	3.031
TAL	NEUg	SUPg	−5.559
TAL	NEUg	PROg	1.99
TAL	SUPg	PROg	6.867

Abbreviations: NEUg = neutral group; SUPg = supinated group; PROg = pronated group; BMI: body mass index; SIN = basal condition sport shoes; SUP = supinated orthoses; PRO = pronated orthoses; TAL = heel lift orthoses.

**Table 5:** Baseline EMG signals mean values of each group without interventions.

NEUg SIN Mean (mV) ± SD (95% CI)	SUPg SIN Mean (mV) ± SD (95% CI)	PROg SIN Mean (mV) ± SD (95% CI)	*p*-Value SIN NEUg vs. SUPg	*p*-Value SIN NEUg vs. PROg	*p*-Value SIN SUPg vs. PROg
87.58 ± 4.81 (85.16–90)	97.17 ± 4.3 (95.35–98.99)	86.55 ± 4.5 (84.32–88.78)	<0.05 *	1	<0.05 *

Abbreviations: SD = standard deviation; CI = confidence interval; mV = millivolts; SIN = basal condition sport shoes; SUP = supinated orthoses; PRO = pronated orthoses; TAL = heel lift orthoses; *p*-value = level of significance; *p* < 0.05 * (with a 95% confidence interval) was considered statistically significant.

**Table 6:** EMG signal amplitudes of the tensor fascia latae mean muscle activity for each group between the different study interventions.

	SIN Mean (mV) ± SD (95% CI)	SUP Mean (mV) ± SD (95% CI)	PRO Mean (mV) ± SD (95% CI)	TAL Mean (mV) ± SD (95% CI)	*p*-Value SIN vs. SUP	*p*-Value SIN vs. PRO	*p* -Value SIN vs. TAL	*p*-Value SUP vs. PRO	*p* -Value SUP vs. TAL	*p*-Value PRO vs. TAL
Neutral group (NEUg)	87.58 ± 4.81 (85.16–90)	88.16 ± 4.76 (86.14–90.18)	74.69 ± 3.77 (73.09–76.29)	83.64 ± 3.4 (76.61–80.49)	1	<0.001 **	0.86	0.08	1	1
Supinated group (SUPg)	97.17 ± 4.3 (95.35–98.99)	105.22 ± 4.9 (103.14–107.3)	90.96 ± 4 (89.27–92.65)	107.02 ± 4.88 (107.2–111.16)	<0.05 *	<0.001 **	<0.05 *	<0.05 *	1	<0.001 **
Pronated group (PROg)	86.55 ± 4.5 (84.32–88.78)	77.81 ± 3.8 (75.93–79.69)	78.58 ± 4.2 (76.5–80.66)	76.62 ± 3.7 (74.79–78.45)	1	1	1	1	1	1

Abbreviations: SD = standard deviation; CI = confidence interval; mV = millivolts; SIN = basal condition sport shoes; SUP = supinated orthoses; PRO = pronated orthoses; TAL = heel lift orthoses; *p*-value = level of significance; *p* < 0.05 * (with a 95% confidence interval) was considered statistically significant; *p* < 0.001 ** (with a 95% confidence interval) was considered strong statistically significant.

## Data Availability

The data from this article will be made available by the authors on reasonable request.
